# Bone transcriptomics reveals that juzentaihoto remodels and activates multiple pathways in Klotho-deficient mice

**DOI:** 10.1016/j.bonr.2026.101936

**Published:** 2026-07-02

**Authors:** Akiko Maruko, Kenshiro Oshima, Akinori Nishi, Yoshinori Kobayashi, Norihiro Okada

**Affiliations:** a Department of Pharmacognosy, School of Pharmacy, Kitasato University, Minato-ku, Tokyo, 108-8641, Japan; b TSUMURA Advanced Technology Research Laboratories, TSUMURA & CO., Ibaraki, 300-1192, Japan; c Oriental Medicine Research Center, School of Pharmacy, Kitasato University, Minato-ku, Tokyo, 108-8641, Japan

**Keywords:** Osteoporosis, Juzentaihoto, Bone remodeling, RNA sequencing, Klotho mouse, Aging-related bone loss

## Abstract

**Background:**

Aging-related osteoporosis arises from an imbalance between bone formation and resorption. The traditional Japanese Kampo formula juzentaihoto (JTT) is used in the clinic to improve imbalances in systemic homeostasis, especially in elderly individuals; however, its effects on age-associated bone loss remain unclear. Here, we confirmed the effect of JTT on the osteoporotic phenotype in a model of aging, and explored its underlying mechanisms through transcriptomic analysis.

**Methods:**

Klotho-deficient mice, which have a shortened lifespan and display an aging-like phenotype that includes osteoporosis, were analyzed. Femoral bone mineral density (BMD) and cortical parameters were assessed by micro–computed tomography. RNA sequencing of femoral tissue was performed to identify differentially expressed genes in response to JTT administration, followed by Gene Ontology analysis and validation by RT–qPCR.

**Results:**

JTT treatment attenuated the decreased in BMD and cortical bone mass and increased cortical void volume, suggesting the potentially reflecting a reactivation of bone remodeling. Consistent with these findings, JTT restored the expression of genes involved in collagen biosynthesis, osteoblast differentiation, mineralization, and transcription factor NFATc1-centered osteoclast maturation. JTT also attenuated aberrant neurotransmitter-related signaling and induced the expression of genes encoding antioxidant enzymes, suggesting additional supportive effects on skeletal homeostasis. These changes occurred without recovery of the Klotho–fibroblast growth factor 23 (FGF23) or vitamin D pathways.

**Conclusions:**

JTT ameliorates low-turnover osteoporosis in Klotho-deficient mice by modulating multiple regulatory pathways that collectively promote bone remodeling. These multitarget actions highlight the therapeutic potential of JTT for age-related bone fragility and other conditions characterized by impaired bone formation.

## Highlights

• Juzentaihoto (JTT) ameliorates low turnover osteoporosis in Klotho-deficient mice.
• JTT restores expression of collagen and bone formation-related genes.
• JTT modulates NFATc1-centered osteoclast and osteoblast pathways.
• JTT induces antioxidant enzyme genes that may support skeletal homeostasis.

## 1 Introduction

Osteoporosis is a skeletal fragility disorder characterized by reduced bone mineral density and bone mass and deterioration of the bone microarchitecture that particularly affects collagen-based structures. Under physiological conditions, bone remodeling and bone metabolism are maintained in a tightly regulated balance between osteoclastic bone resorption and osteoblastic bone formation. However, under specific pathological conditions, including aging, menopause, medication exposure, and secondary diseases, this balance is disrupted, leading to excessive bone resorption relative to bone formation and ultimately resulting in osteoporosis and related skeletal disorders (Feng and Teitelbaum, 2013).

Osteoporosis is a major risk factor for fractures and is associated with reduced quality of life and impaired daily activities, underscoring the importance of effective prevention and treatment strategies. In humans, postmenopausal osteoporosis, which predominantly affects women, is driven by estrogen deficiency, which increases bone resorption and subsequent bone formation, resulting in a high bone turnover phenotype (Eastell et al., 2016; Committee for Developing Guidelines for the Prevention and Treatment of Osteoporosis, 2025). In contrast, osteoporosis in males is typically characterized by age-related decreases in bone formation and reduced bone remodeling activity (Compston et al., 2019). Although the prevalence of osteoporosis is lower in men than in women, men exhibit higher one-year mortality rates following proximal femoral fractures (Committee for Developing Guidelines for the Prevention and Treatment of Osteoporosis, 2025; Sing et al., 2023), highlighting the clinical importance of prevention and treatment in both sexes. Importantly, improving bone strength requires not only increases in bone mineral density but also broader enhancements in bone quality, including bone turnover dynamics and the degree of bone matrix mineralization.

Klotho was originally identified as an anti-aging factor, and klotho knockout mice (Klotho mice) exhibit multiple phenotypes characteristic of human aging, including a shortened lifespan, arteriosclerosis, emphysema, and skin atrophy (Kuro-o et al., 1997). The Klotho protein is predominantly expressed in the kidney, brain choroid plexus, and parathyroid gland, and in the kidney, it plays a central role in the regulation of calcium, vitamin D, and phosphate metabolism. To perform these functions, Klotho forms a complex with the bone-derived endocrine hormone fibroblast growth factor 23 (FGF23) and its receptor (FGFR) (Quarles, 2012; Gutierrez, 2013; Erben and Andrukhova, 2017). In the parathyroid gland, FGF23-Klotho signaling suppresses the synthesis and secretion of parathyroid hormone (PTH) (Andrukhova et al., 2016). Klotho is required for maintaining bone quality; thus, klotho-deficient mice exhibit reduced numbers of osteoclasts and osteoblasts, reduced bone remodeling activity, and decreased bone mass, making them valuable models of human senile osteoporosis (Kuro-o et al., 1997; Kawaguchi et al., 1999; Kawaguchi, 2006). Compared to wild-type (WT) mice, the phenotypic and skeletal divergence in klotho-deficient mice is remarkably profound. By 7 weeks of age, these mice exhibit severe growth retardation, with their body weight dropping to approximately one-third to one-half of that of WT controls (Kuro-o et al., 1997; Yamashita et al., 1998). Klotho-deficient mice display severe osteopenia, featuring a dramatic reduction in bone mineral density (BMD; up to 35.9% in the diaphysis) and an approximately 40% reduction in cortical bone thickness compared to WT mice (Kawaguchi et al., 1999).

Current standard treatments for osteoporosis include a variety of pharmacological interventions such as hormone therapy, calcium and vitamin D supplementation, anti-resorptive agents (e.g., bisphosphonates and denosumab), and bone anabolic agents (e.g., teriparatide and romosozumab). However, long-term use of bisphosphonates is associated with adverse events such as atypical femoral fractures and is often limited by complex dosing regimens. In addition, suboptimal medication adherence remains a significant clinical challenge across osteoporosis therapies (Committee for Developing Guidelines for the Prevention and Treatment of Osteoporosis, 2025; Mazziotti et al., 2025). Furthermore, the high prevalence of comorbidities in older adults, particularly reduced renal function, which directly affects drug metabolism, requires careful dose selection and monitoring, underscoring additional clinical challenges that remain in osteoporosis management (Committee for Developing Guidelines for the Prevention and Treatment of Osteoporosis, 2025).

Kampo medicine (Japanese traditional herbal medicine) has been integrated into modern medical practice and is widely used in Japan to improve patients' quality of life. Unlike most Western medicines, which typically consist of a single active compound with rigorously defined pharmacological effects, Kampo formulas are composed of multiple herbs. The complex interactions among these components can mitigate the adverse effects of individual herbs and, in some cases, generate synergistic effects that increase therapeutic efficacy (Hosoya and Yamamura, 1988; Kiyohara et al., 2004). Juzentaihoto (JTT) is a representative “nourishing” Kampo formula composed of ten medicinal herbs that has traditionally been used to improve weakness, deficiency states, and disturbances in physiological homeostasis (Saiki, 2000). JTT has diverse beneficial effects, including supporting recovery from illness; improving symptoms such as anemia, anorexia, chronic fatigue, rheumatoid arthritis, and dry or scaly skin; and reducing the risk of infection or cancer metastasis in immunocompromised individuals (Saiki, 2000; Amitani et al., 2015). In traditional Kampo theory, JTT is a formula that is considered to replenish the dual deficiency of “qi” (vital energy) and blood, thereby restoring vitality in elderly or debilitated individuals.

Evidence supports the efficacy of JTT in mouse models of osteoporosis. In these models, JTT restored bone mineral density and the expression of bone formation markers (G.-B and J.-C, 2002) and suppressed bone deterioration by lowering serum PTH levels and increasing the femoral calcium and phosphorus content (Chen et al., 2005). Takeda et al. reported that JTT inhibits periodontal bone resorption by suppressing osteoclast formation in a periodontitis rat model (Takeda et al., 2014). While these previous studies successfully established the therapeutic potential of JTT by evaluating individual phenotypic and physiological parameters, its underlying molecular mechanisms at the gene level remain largely uncharacterized. Because Kampo formulas contain multiple herbal components, their actions often involve complex and interconnected molecular pathways. A comprehensive, genome-wide transcriptomic approach is therefore useful for capturing the coordinated changes across various biological pathways induced by JTT.

In the present study, we provide the first detailed transcriptomic characterization of JTT's multi-pathway effects by combining bone structural analysis with RNA sequencing in a mouse model of senile osteoporosis. JTT broadly activated pathways related to both bone formation and resorption, suggesting its potential to ameliorate age-related impairments in bone metabolism. These findings provide foundational evidence supporting the therapeutic potential of JTT for age-associated bone disorders.

## 2 Materials and methods

### 2.1 Juzentaihoto (JTT)

The extract powder of Juzentaihoto (JTT; TJ-48) was supplied as a preservative-free, pharmaceutical-grade powder by TSUMURA & CO. (Tokyo, Japan). This Kampo formulation was reviewed and evaluated by the Pharmaceuticals and Medical Devices Agency (PMDA) and approved by the Minister of Health, Labour and Welfare of Japan in 1986 (approval no. 16100AMZ01124000). JTT extract powder was prepared by spray-drying a hot-water extract obtained from a mixture of the following 10 crude drugs at the ratios indicated (parts by weight), using the English nomenclature of the 18th edition of the Japanese pharmacopoeia: Astragalus root (3.0), cinnamon bark (3.0), Rehmannia root (3.0), peony root (3.0), Cnidium rhizome (3.0), Atractylodes lancea Rhizome (3.0), Japanese Angelica root (3.0), ginseng (3.0), Poria Sclerotium (3.0), and Glycyrrhiza (1.5). All crude drugs are listed in the Japanese Pharmacopoeia, 18th edition (2021). The origin and species of each drug, the contents of the characteristic ingredients, and other pharmaceutical-grade qualities of JTT are strictly controlled, as it is an ethical drug approved by the Ministry of Health, Welfare and Labor of Japan.

### 2.2 Animals

α-Klotho knockout (KO/Jcl) mice (klotho mice) were generated by targeted disruption of the α-Klotho gene on a C57BL/6JJcl background. Wild-type C57BL/6JJcl (WT) mice were used as controls. Male klotho mice and WT mice (3 weeks old) were acclimated for 0.5 weeks in a vinyl isolator, during which time they were given radiation-sterilized water and a CE-2 diet (CLEA Japan, Inc., Tokyo, Japan) ad libitum. The mice were housed in a vivarium maintained at a constant temperature (23 ± 2 °C) and humidity (55 ± 10%) and under a 12-h light/dark cycle (lights on at 07:00). After acclimation, the klotho mice were divided into two groups. One group of klotho mice was fed CE-2 containing 0.5% (w/w) JTT (referred to as KL+), whereas the other klotho group (KL−) and the WT group were fed CE-2 from 3.5 weeks of age for 3.5 weeks. At 7 weeks of age, five mice from each group were anesthetized with isoflurane. The brain, femur, bone marrow, and skeletal muscle were collected from the same animals. The isolated organs were immediately placed in RNAlater (Thermo Fisher Scientific, Waltham, MA) at room temperature and then stored at 4 °C for 12–14 days. Afterward, the samples were stored frozen at −25 °C for subsequent RNA extraction. Kidney samples were obtained from an independent cohort of mice (n = 4 per group) that were bred, housed, treated, and sampled at different time points under identical experimental conditions and using the same protocols described above.

For bone structure analysis, 7-week-old male KL− and KL+ mice (n = 5 per group), which were maintained under identical conditions in separate cohorts at different times, were anesthetized with isoflurane, and their femurs were collected. After the surrounding soft tissues were removed, the femurs were immediately fixed in 70% ethanol and processed for bone structure analysis.

All of the above breeding and dissections were performed at CLEA Japan, Inc., Tokyo.

### 2.3 Bone structure analysis

Femurs were subjected to micro-computed tomography (micro-CT) using a ScanXmate-L090H system (Comscantecno, Kanagawa, Japan) to measure bone mineral density (BMD; mg/cm^3^) and cortical bone parameters. The micro-CT scans were performed under the following conditions: voltage, 75 kV; current, 100 μA; voxel size/resolution of 28.037 μm/pixel and thickness of 28.037 μm. For analysis, each femur was divided into quintiles along its length, and each quintile was scanned and reconstructed into a three-dimensional image using TRI/3D-BON (RATOC System Engineering, Tokyo, Japan).

To measure the cortical bone parameters, a site corresponding to 25% of the total femur length from the distal end of the femur was subjected to the following measurements using a Scan Xmate-L090 and TRI/3D-BON (voltage, 75 kV; current, 100 μA; voxel size/resolution of 9.097 μm/pixel and thickness of 9.097 μm): cortical bone area (Ct.Ar), medullary area (Me.Ar), total cross section area inside the periosteal envelope (Tt.Ar), cortical area fraction (Ct.At/Tt.Ar), average cortical thickness (Ct.Th), total pore volume (Po.V), cortical porosity (Ct.Po), cortical bone density (Ct.B.Dn), periosteal perimeter (Ps.Pm), and endoortical perimeter (Ec.Pm). All of the above bone structure measurements were performed at KUREHA SPECIAL LABORATORY Co., Ltd., Fukushima, Japan.

### 2.4 RNA extraction and RNA-seq

Before RNA extraction, surrounding skeletal muscle was removed from each frozen femur, and the metaphyseal/epiphyseal regions were cut off and discarded. The remaining diaphysis was then split longitudinally with a scalpel, and the bone marrow was thoroughly scraped out. This isolated cortical bone tissue is expected to encompass the resident cell populations embedded within or residing on the bone matrix (primarily osteocytes, osteoblasts, and osteoclasts), as well as intracortical endothelial cells and traversing peripheral nerve fibers. Both the cortical bone tissue of the diaphysis and the harvested bone marrow were used for subsequent RNA isolation. Femur, skeletal muscle, and kidney samples were cut into small pieces (∼30 mg each) using scissors. Brain samples were prepared from a single cerebral hemisphere.

RNA extraction was performed on individual tissue samples taken from each femoral diaphysis, skeletal muscle, marrow, kidney, and brain sample using a Pure Link RNA Mini Kit (Invitrogen, Carlsbad, CA). Briefly, tissues were lysed in 300 μL of lysis buffer and 450 μL of TRIzol-LS Reagent (Thermo Fisher Scientific) and homogenized on ice using a Wheaton Dounce tissue grinder. The homogenates were then incubated at room temperature for 10 min and centrifuged at 12,000 ×*g* for 15 min. The resulting supernatants were treated with DNase, and total RNA was subsequently purified using spin columns according to the manufacturer's instructions. The quality of the total RNA was examined with a Qubit 3.0 (Thermo Fisher Scientific) and 4200TapeStation system (Agilent Technologies, Santa Clara, CA). Each total RNA sample had a RIN > 7, indicating that it was of sufficient quality to prepare sequencing libraries.

Total RNA was used for RNA-seq. mRNA library construction was performed by using a TruSeq Stranded mRNA Sample Prep kit (Illumina, San Diego, CA). Libraries were sequenced on the Illumina NovaSeq 6000 platform, and 35 million (150-bp paired-end) reads were generated. RNA-seq was performed at Takara Bio Inc., Shiga, Japan.

### 2.5 Quality control and filtering of RNA-seq data and mapping analysis

To preprocess the sequencing data, cutadapt v.1.16 (Martin, 2011) was used to remove Illumina adapter sequences, followed by removal of the poly(A) sequence using fastx_clipper software in the fastx toolkit software package v.0.0.14 (http://hannonlab.cshl.edu/fastx_toolkit/). To remove low-quality bases or sequences, we trimmed the sequences using fastq_quality_trimmer software (parameters: -t 20 -l 30 -Q 33) and fastq_quality_filter software (parameters: -q 20 -p 80 -Q 33), both of which are included in the fastx toolkit. During the above processing, any reads in which one of the pairs was missing were removed using Trimmomatic v.0.38 (Bolger et al., 2014). Afterward, reads containing mouse rRNA, tRNA, or phiX sequences (the last of which is the control sequence from Illumina) were removed using Bowtie 2 v. 2.4.4 (Langmead and Salzberg, 2012). We then carried out a second processing step to remove any unpaired reads using bam2fastq. After these filtering steps were completed, 20 million reads of each of the forward and reverse sequences per sample were mapped to the mouse genome sequence build GRCm38 using HISAT2 v2.2.1 (Kim et al., 2019). The mouse genome sequence was downloaded from Ensembl genome (release 109). Multiple mapped reads were removed using samtools v. 1.23 (parameters: samtools view -q 4). Uniquely mapped reads were counted by gene annotation (Ensembl release 109) using featureCounts v.2.0.3. The raw read counts were normalized using the trimmed mean of M values (TMM) method with the edgeR (Robinson et al., 2010) library in R software (version 4.5.1). To identify differentially expressed genes (DEGs), we employed a generalized linear model (GLM). Statistical significance was assessed using the likelihood ratio test (LRT) after common and tagwise dispersions were estimated. Principal component analysis (PCA) plots were generated for genes with expression level TMM values >1 using the ggplot2 library in R.

### 2.6 Analysis of differentially expressed genes (DEGs)

Genes whose expression significantly differed (*P* < 0.05) between the two groups were identified using edgeR. *P* values for the differential expression of genes were determined via the likelihood ratio test. DEGs were subjected to Gene Ontology (GO) biological analysis (http://www.geneontology.org/) and Kyoto Encyclopedia of Genes and Genomes (KEGG) analysis (https://www.genome.jp/kegg/) using DAVID (Sherman et al., 2022), a functional annotation online database (v.2024q4) (https://david.ncifcrf.gov/). A likelihood ratio test was applied to identify significant GO terms, and the threshold of significance was defined by the *P* value. To evaluate and validate the enriched GO terms obtained from DAVID, gene set enrichment analysis (GSEA) (Mootha et al., 2003; Subramanian et al., 2005) was performed using the clusterProfiler package v. 4.14.6. For the GSEA, all detected genes were ranked on the basis of their log fold -change (logFC) values. Enriched functions were identified using the GO biological process database. The magnitude of enrichment for each gene set was quantified using the normalized enrichment score (NES), and statistical significance was defined by the adjusted *P* value.

### 2.7 Quantitative real-time reverse transcription polymerase chain reaction (RT–qPCR) and data analysis

To analyze mRNA expression, total RNA was reverse-transcribed into complementary DNA (cDNA). Quantitative reverse transcription–PCR (RT–qPCR) was performed using a StepOne Real-Time PCR system (Applied Biosystems) with a Power SYBR™ Green RNA-to-CT 1-Step Kit (Thermo Fisher Scientific). PCR amplification and data acquisition were carried out using StepOne software v2.3 and Opticon Monitor software version 3.1 (Bio-Rad, Hercules, CA). The expression levels of target genes were normalized to that of *Gapdh*, and relative expression was calculated via the 2^−ΔΔCt^ method. The sequences of the primers used for RT–qPCR are listed in Table 1. For *Col1a1*, two independent primer sets (Col1a1_1 and Col1a1_2) were used to validate expression robustness.

**Table 1:** List of primer sequences used for RT–qPCR.

Primer name	Forward primer (5′ to 3′)	Reverse primer (5′ to 3′)
*Bglap*	TTTCTGCTCACTCTGCTGACC	GTAGGCGGTCTTCAAGCCAT
*Col1a1_1*	AGCTGCATACACAATGGCCT	GGGTTGGGACAGTCCAGTTC
*Col1a1_2*	CCCAGTGGCGGTTATGACTT	CTCAAGGTCACGGTCACGAA
*Col1a2*	TCCAGGCCCAACCTGTAAAC	GGCTGCCACCATTGATAGTCT
*Csf1*	AAGACAACACCCCCAATGCT	GCTCCTCATAGTCCTTGGTGA
*Ctsk*	CAGTGTTGGTGGTGGGCTAT	CATGTTGGTAATGCCGCAGG
*Dcstamp*	TGATGGGTGTGAACCACGAG	GCCCTTCCACAATGCCAAAG
*Gapdh*	TGATGGGTGTGAACCACGAG	GCCCTTCCACAATGCCAAAG
*Gpx1*	AGTCCACCGTGTATGCCTTC	CCTCAGAGAGACGCGACATT
*Itgb3*	GACTCAAGCAACGTCCTCCA	TCCAATCTTGAGGCCCACAC
*Nfatc1*	GGAAGAACACCAGGGTGAGG	CACTCGATAGGGTTCGAGGC
*Oscar*	ATCAGCTCCCCAGACCATCA	CGCGGTACAGTGCAAAACTC
*Prdx2*	TGACCTTGGAAAGGCAAGACA	CAGGAGCCGACTTTCCGATT
*Runx1*	GAACCACAAGTTGGGTAGCCT	CGAAAGCCTGTGGTTTGCAT
*Sod1*	GGAAGCATGGCGATGAAAGC	CCCCATACTGATGGACGTGG
*Sparc*	ACCTGGACTACATCGGACCA	TCAGTGAGGAGGTTGTTGCC

Statistical analyses of the RT–qPCR data were performed using R software. Comparisons among three groups were conducted with appropriate multiple-comparison procedures. Data normality was assessed using the Shapiro–Wilk test, and homogeneity of variance was evaluated using Levene's test. When the data satisfied both normality and homogeneity of variance, one-way ANOVA was applied, followed by Tukey's honestly significant difference (HSD) test. When normality was met but variances were unequal, Welch's ANOVA was performed, followed by the Games–Howell post hoc test. When the data did not meet the assumption of normality, the Kruskal–Wallis test was used, followed by Dunn's multiple-comparison test. A *p*-value < 0.05 was considered to indicate statistical significance.

## 3 Results

### 3.1 JTT attenuates age-related decreases in femoral BMD and cortical thickness in Klotho-deficient mice

**Fig. 1:**
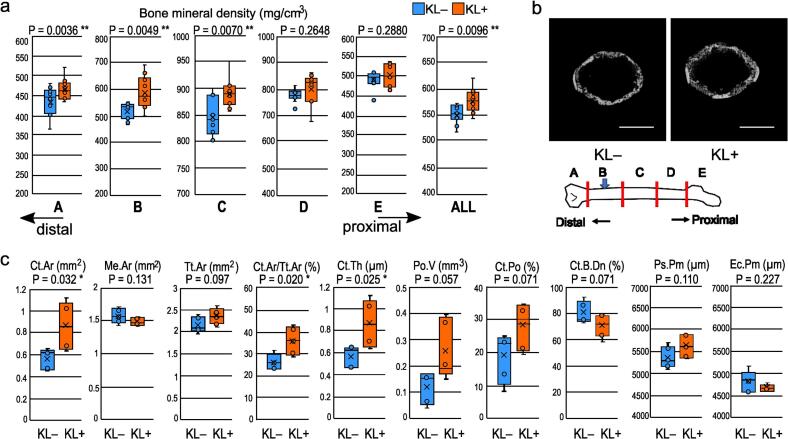
JTT improved bone mineral density and cortical bone thickness in klotho mice. (a) Bone mineral density (BMD) of 7-week-old klotho mice (KL-) and JTT-treated klotho mice (KL+) (n = 5 in each group). The femur was divided into five equal segments (A–E) from the distal to the proximal end, as illustrated in (b). (b) 2D micro-CT cross-sectional images of the distal diaphysis of the femur (central section B, blue arrow). Representative images from five independent experiments are shown. Scale bar = 1 mm. (c) Analysis of cortical bone parameters in segment B from KL− and KL+ mice. *P < 0.05 by two-sided Welch's *t*-test. Ct.Ar, cortical bone area; Me.Ar, medullary area; Tt.Ar, total cross section area inside the periosteal envelope; Ct.At/Tt.Ar, cortical area fraction; Ct.Th, average cortical thickness; Po.V, total pore volume; Ct.Po, cortical porosity; Ct.B.Dn, cortical bone density; Ps.Pm, periosteal perimeter; Ec.Pm, endoortical perimeter. Box plots indicate medians with interquartile ranges; x indicates the mean; whiskers indicate 1.5 times the interquartile distance.

To analyze the effect of JTT on osteoporosis, we first analyzed its effects on bone structure. After 3.5 weeks of JTT treatment, femur bones were isolated from each mouse, and the BMD of each femur was determined by micro-CT (Fig. 1a) to compare the BMDs of KL− and KL+ mice. Micro-CT analysis revealed an increase in BMD in KL+ mice, especially in the distal femur (i.e., positions A, B, and C in Fig. 1b). Next, we performed cortical bone parameter analysis at position B (Fig. 1b, c). CT images of the femur revealed that the cortical bone was thickened by JTT administration (Fig. 1b, Supplementary Fig. 1). As shown in Fig. 1c, the cortical bone volume fraction (Ct.Ar, Ct.Ar/Tt.Ar) and thickness (Ct.Th) significantly increased, whereas the void (Po.V) slightly increased in KL+ mice compared with those in KL− mice. As such, there was also a slight increase in the cortical porosity (Ct.Po) and a decrease in cortical bone density. The Mv, Tt.Ar, Ps.Pm, and Ec.Pm were not altered by JTT. Klotho mice exhibit low turnover, similar to that observed in osteopenia, because of decreased osteoblast and osteoclast differentiation (Kawaguchi et al., 1999). Therefore, the administration of JTT may restore both bone formation and resorption, potentially affecting bone remodeling patterns or influencing cortical porosity in klotho mice.

### 3.2 JTT attenuates the aging-associated downregulation of bone-related gene sets

**Fig. 2:**
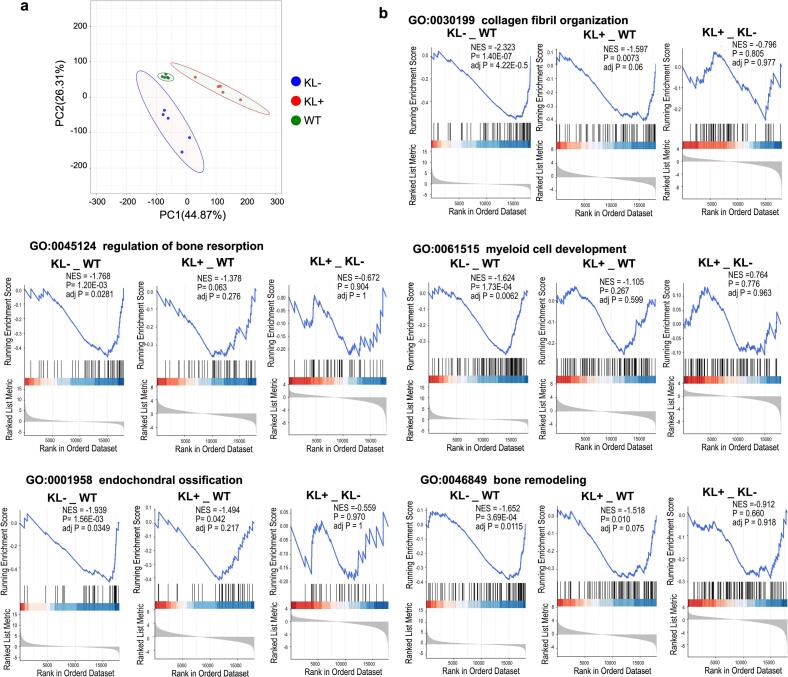
Gene set enrichment analysis (GSEA) of bone-related pathways in the femurs of Klotho mice. (a) Principal component analysis (PCA) of femoral RNA-seq data from KL−, KL+, and wild-type (WT) mice revealed that each group formed a distinct cluster. (b) GSEA was performed using RNA-seq data from femoral tissue, and bone-related Gene Ontology (GO) terms that were significantly enriched (adjusted *P* < 0.05) in the KL− versus WT comparison, including “collagen fibril organization,” “myeloid cell development,” “bone remodeling,” “regulation of bone resorption,” and “endochondral ossification”, were extracted for visualization. Enrichment plots are shown for the KL− versus WT (left), KL+ versus WT (center), and KL+ versus KL− (right) comparisons. The y-axis represents the enrichment score, and the x-axis shows the ranked list of genes. The vertical bars indicate the positions of the genes belonging to each gene set, and the running enrichment score (blue line) reflects the degree of enrichment across the ranked list. The normalized enrichment score (NES), *P* value, and adjusted *P*-value (adj P) are shown in each plot.

To investigate which genes involved in bone formation and resorption are altered by JTT, RNA-seq analysis was performed on the femoral shafts of KL−, KL+, and wild-type (WT) mice. Principal component analysis (PCA) was performed using genes whose TMM values were greater than 1 (Fig. 2A). PCA with 95% confidence ellipses (stat_ellipse, level = 0.95) revealed that all the replicates clustered tightly within their respective groups, with no apparent outliers. The three groups were also clearly separated along the principal components.

To capture global transcriptional differences between two groups, we performed GSEA and examined GO terms related to bone biology (Fig. 2b). In the comparison between WT and KL− mice, several bone-related GO terms with significantly altered adjusted *P* values, including “collagen fibril organization,” “myeloid cell development,” “bone remodeling,” “regulation of bone resorption,” and “endochondral ossification”, exhibited a negative bias in the KL− group (Fig. 2b left). Although the KL+ group also exhibited a negative bias in the KL+ vs. WT comparison, the adjusted P values indicated that the differences were not significant, suggesting that JTT administration mitigated these negative biases. These findings indicate that the expression of genes related to both bone formation and bone resorption was reduced in the KL− group but was partially restored by JTT treatment. However, in the direct comparison between KL− and KL+ mice, these GO terms showed minimal differences (Fig. 2b right).

### 3.3 GO-based profiling of KL− mice and JTT-mediated restoration in the femur and associated tissues

**Fig. 3:**
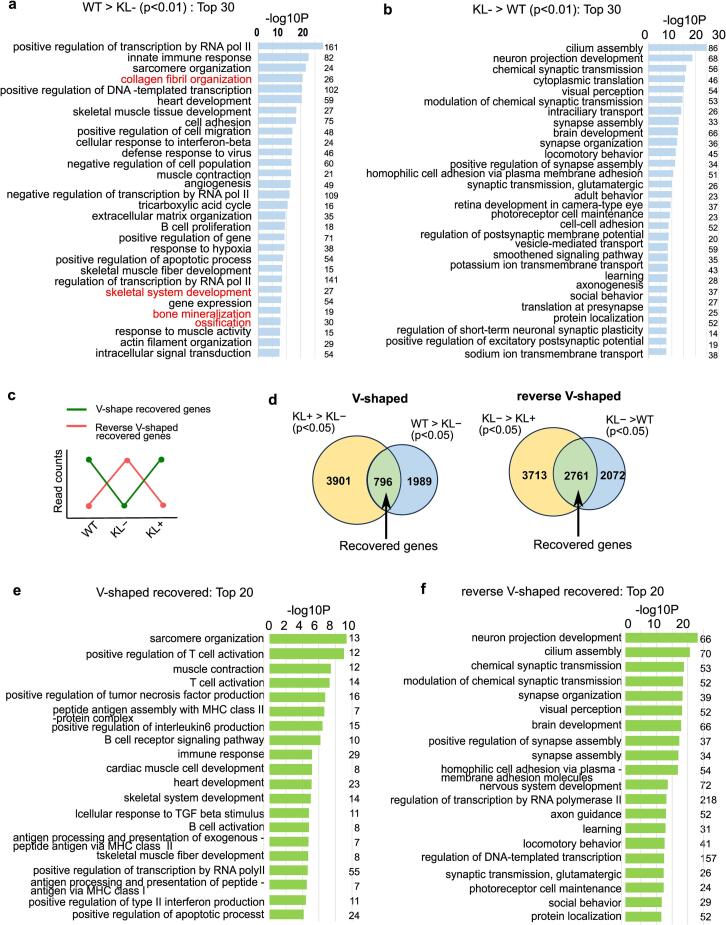
Gene Ontology (GO) enrichment analysis of the differentially expressed genes (DEGs) and JTT-recovered genes in femoral samples. (a, b) The top 30 GO terms associated with biological processes enriched in the DEGs (*P* < 0.01) between KL− and WT mice, as determined via the likelihood ratio test. (a) WT vs. KL− and (b) KL− vs. WT comparisons. Bone-related GO terms are highlighted in red. (c) Genes whose expression was significantly downregulated in KL− mice compared with WT mice and whose expression was significantly upregulated in KL+ mice (green line) are referred to as V-shaped recovered genes. Genes significantly upregulated in KL− mice compared with WT mice and that were significantly downregulated in KL+ mice (red line) are referred to as reverse V-shaped recovery genes. (d) Venn diagram showing the number of genes whose expression significantly differed (*P* < 0.05) between the KL− vs. WT and KL− vs. KL+ comparisons. Overlapping genes indicate V-shaped recovery genes (left) and reverse V-shaped recovery genes (right). (e) The top 20 significant GO terms associated with V-shaped recovered genes. (f) The top 20 significant GO terms associated with the reverse V-shaped recovery genes. In (a), (b), (e) and (f), gene counts are shown to the right of each term. The negative log of the *P* value is plotted on the x axis.

Next, we identified differentially expressed genes (DEGs) between WT and KL− mice using a threshold of *P* < 0.01 and performed GO analysis by using the DAVID functional annotation tool (Fig. 3a, b). Compared with those in WT mice, genes significantly downregulated in KL− mice (1776 genes) were enriched in bone-related GO terms such as “collagen fibril organization,” “bone mineralization,” and “ossification” (Fig. 3a, red letters). In contrast, genes upregulated in KL− mice (3744 genes) were strongly enriched for neuronal GO terms, including “neuron projection development,” “synapse assembly,” and “brain development” (Fig. 3b). We then extracted JTT-responsive recovery genes using DEGs on the basis of *P* < 0.05. This threshold was adopted to maximize sensitivity and comprehensively profile the multi-component regulatory network of JTT, ensuring that moderate yet biologically significant changes in the recovery pathway would not be overlooked. Genes whose expression was downregulated in KL− mice compared with that in WT mice but whose expression was significantly upregulated by JTT treatment were referred to as V-shaped recovery genes (Fig. 3c, green line). Conversely, genes whose expression was upregulated in KL− mice compared with that in WT mice but whose expression was significantly downregulated by JTT were referred to as reverse V-shaped recovery genes (Fig. 3c, red line). As shown in the Venn diagram, 796 V-shaped and 2761 reverse V-shaped recovery genes were identified (Fig. 3d). GO analysis of these recovery genes revealed that the most terms enriched in the V-shaped recovery genes were not related to bone; instead, they were dominated by muscle-associated terms such as “sarcomere organization” and “muscle contraction,” as well as immune-related terms such as “T-cell activation,” “B-cell activation,” and “immune response” (Fig. 3e). Nevertheless, bone-related GO terms such as “collagen fibril organization,” “osteoclast differentiation,” and “ossification” were still detected at the threshold of *P* < 0.01 (Table S1). Consistent with these findings, KEGG pathway analysis also revealed muscle- and immune-related pathways, including “cytoskeleton in muscle cells,” “human T-cell leukemia virus 1 infection,” and “antigen processing and presentation” (Table S1).

The observation that skeletal muscle- and immune-related genes appeared among the recovery genes in femoral RNA suggested the possibility of contamination, as complete removal of bone marrow and surrounding connective tissues during manual dissection of the femur is technically difficult. However, it is important to note that our femoral RNA-seq analysis reflects the whole cortical bone microenvironment; the samples inherently contain not only osteocytes but also resident cells such as endosteal osteoblasts and osteoclast-lineage cells. To address the concern regarding the potential contribution of contaminating marrow or muscle components, we also analyzed RNA-seq data from femoral muscle and bone marrow (Supplemental Fig. 2). PCA revealed no clear separation between the KL− and KL+ muscle samples, whereas the bone marrow samples of the three groups were well separated (Supplemental Fig. 2a). When PCA was performed using genes commonly expressed across the femur, muscle, and bone marrow, the three tissues exhibited distinct clustering (Supplemental Fig. 2b). To further verify the efficiency of bone marrow removal from the femoral shaft, we compared the expression levels of well-established bone- and marrow-specific marker genes between the femur and bone marrow samples (Supplemental Fig. 2c). The isolated femur samples exhibited robust expression of the osteocyte markers Sost and Dmp1. Conversely, the expression of pan-bone marrow, microenvironmental, and hemoglobin markers (*Ptprc*, *Cxcl12*, and *Hbb-bs*) was drastically and significantly lower in the femur than in the bone marrow cavity. Although low-level baseline expressions of these marrow-associated transcripts remained detectable in the femur—reflecting the physiological presence of intracortical elements such as transcortical vessels and resident cells—these clear differences, along with the distinct PCA clustering, confirmed that the mechanical removal of bone marrow was highly successful and the tissue characteristics were robustly preserved.

GO analysis of recovery genes in bone marrow revealed that the top terms enriched in the V-shaped recovery genes closely resembled those identified in the femur, including muscle-related terms such as “sarcomere organization” and “muscle contraction,” as well as immune-related terms such as “immune response” (Supplemental Fig. 2d). Because muscle-related genes were detected in both the femur and bone marrow, we compared the expression patterns of several muscle-specific genes (Raza et al., 2021). The expression profiles of the femur and bone marrow were clearly distinct from those of skeletal muscle (Supplemental Fig. 2e). These findings suggest that the results cannot be explained solely by simple muscle contamination and raise the possibility that JTT may induce myogenic differentiation in bone marrow-derived cells.

Neuronal GO terms were enriched in the reverse V-shaped recovery genes (Fig. 3f and Table S2), similar to the KL− and WT comparison shown in Fig. 3b. Neuronal and synapse-related terms were largely absent from reverse V-shaped recovery genes in bone marrow (Supplemental Fig. 2d) and muscle (data not shown), indicating that these neuronal signatures likely reflect JTT-specific effects within the femur.

### 3.4 JTT restores BMD without affecting the Klotho-FGF pathway

The improvement in BMD in KL-deficient mice by the administration of JTT suggests that the JTT improves BMD through a pathway other than the Klotho-FGF pathway. These findings, however, raise a new question about the extent to which JTT affects the Klotho signaling pathway. The kidney is the most important site of Klotho expression and is also important for the regulation of mineral homeostasis through Klotho-FGF23 signaling. In the kidney, Klotho–FGF23 signaling reduces the expression of sodium-dependent phosphate cotransporter 2a (NaPi2a), thereby inhibiting tubular phosphate reabsorption and decreasing circulating vitamin D levels by inhibiting 1α-hydroxylase (CYP27B1), the enzyme responsible for production of the active molecule 1,25(OH)₂D. The resulting decrease in intestinal phosphate absorption contributes to lower serum phosphate levels. FGF23 secretion from bone is stimulated by 1,25(OH)2D and its receptor (VDR; vitamin D receptor), and FGF23 participates in a bone–kidney feedback loop (Supplementary Fig. 3c) (Quarles, 2012; Gutierrez, 2013; Erben and Andrukhova, 2017).

We examined the expression of Klotho-FGF23-related genes, including *Kl*, *Fgf23*, *Fgfr1*, *Cyp27b1*, *Vdr*, and *Slc34a1* (which encodes NaPi2a), in the RNA-seq data from the kidney (Supplementary Fig. 3a) and femur (Supplementary Fig. 3b). No differences were detected in the expression of *Kl*, *Fgfr1*, *Cyp27b1*, or *Slc34a1* in the kidney or in the expression of *Fgfr1* and *Fgf23* in the femur between KL- and KL+ mice.

The increase in VDR and Fgf23 levels which was likely due to excess vitamin D in klotho mice, remained significantly elevated in KL+ mice compared with WT mice. Therefore, the improvement in BMD observed with JTT treatment is unlikely to have been achieved through vitamin D-related pathways.

### 3.5 Collagen-associated genes are preferentially recovered by JTT

**Fig. 4:**
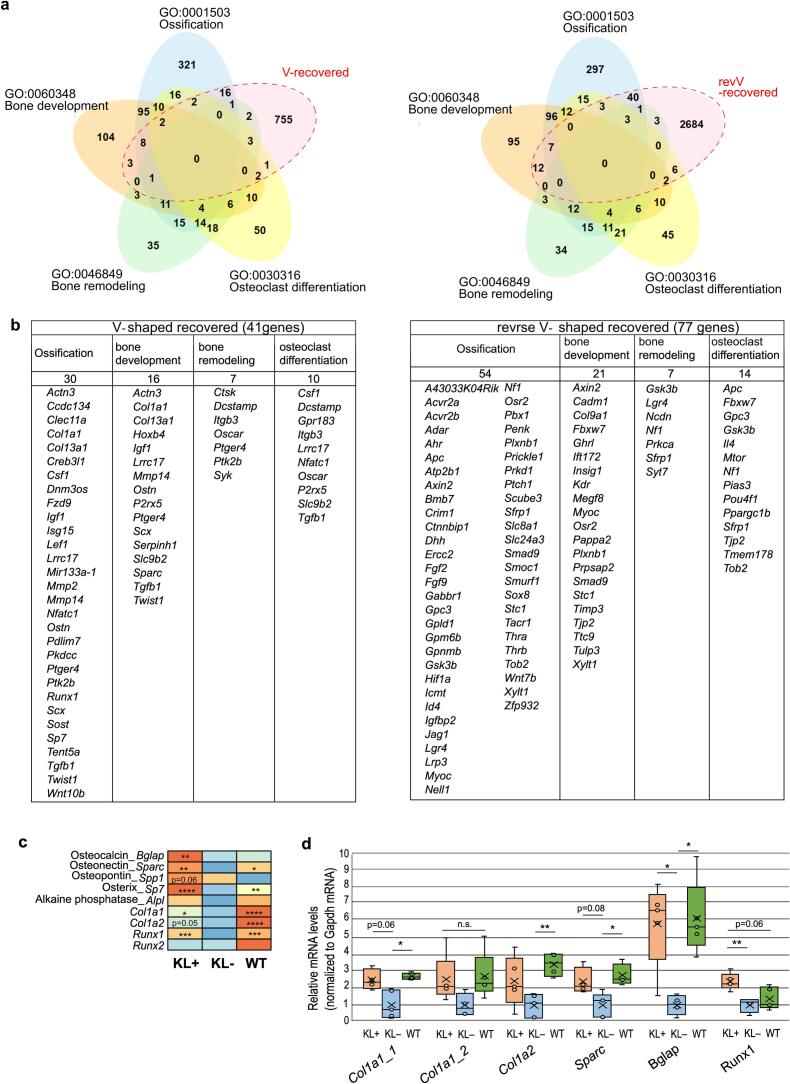
Identification and validation of bone-related genes restored by JTT in femoral samples. (a) Venn diagram showing the overlap of V-shaped or reverse V-shaped recovery genes, with genes included in the bone-related GO terms “ossification,” “bone development,” “bone remodeling,” and “osteoclast differentiation”. V-shaped recovery genes (left); reverse V-shaped recovery genes (right). (b) Lists of genes common to each GO term shown in (a). V-shaped recovery genes (left); reverse V-shaped recovery genes (right). (c) Heatmap of the expression levels of genes associated with bone formation. *P < 0.05, **P < 0.01, ***P < 0.001, ****P < 0.0001 vs. KL− mice, according to the likelihood ratio test. (d) mRNA expression levels of *Col1a1*, *Col1a2*, *Sparc*, *Bglap*, and *Runx1* measured by RT-qPCR are shown as the fold change relative to those in KL− mice (means ± SDs, n = 4–5). *Col1a1_1* and *Col1a1_2* represent two independent primer sets for Col1a1. Box plots display the 25th and 75th percentiles (bottom and top of the box, respectively), the horizontal line within the box represents the median, crosses indicate the mean, and whiskers represent the minimum and maximum values. *Gapdh* was used as an internal control. n.s., not significant; *P < 0.05, **P < 0.01 (statistical tests were performed as described in the Methods).

To clearly identify bone-related genes that were restored by JTT, we examined the overlap between V-shaped or reverse V-shaped recovery genes in the femur and genes included in bone-related GO terms “ossification”, “bone development”, “bone remodeling” and “osteoclast differentiation” (Fig. 4a, b). After removing duplicates, 44 genes that overlapped with the V-shaped recovery genes were identified, and 77 genes that overlapped with the reverse V-shaped recovery genes were identified. The V-shaped recovery genes included *Sparc*, which encodes osteonectin, a protein that is required for bone formation and mineralization; type I collagen genes (*Col1a1*) (Delany and Hankenson, 2009; Rosset and Bradshaw, 2016); collagen turnover-related genes (*Mmp2* and *Mmp14*) (Zigrino et al., 2016); collagen biosynthesis genes (*Creb3l1* and *Serphinh1*) (Boudin and Van Hul, 2017); *Sp7*, which encodes osterix and is essential for the osteoblastic differentiation of bone marrow mesenchymal cells (Chen et al., 2019; Wang et al., 2023); and *Runx1*, a gene important for chondrogenesis (Tang et al., 2020) (Fig. 4b).

Examination of additional genes important for bone formation revealed that the expression of *Bglap* (osteocalcin), *Spp1* (osteopontin), and *Alpl* (alkaline phosphatase) also increased following JTT treatment. Conversely, the expression of *Runx2*, a transcription factor critical for the activation of osteogenic differentiation programs, did not recover (Fig. 4c). qRT–PCR analysis confirmed that several genes exhibited V-shaped recovery patterns (Fig. 4d). Because type I collagen is also expressed in skeletal muscle, we assessed the mRNA levels of type I collagen in the muscle surrounding the femur to exclude the possibility that the JTT-induced recovery reflects muscle-derived collagen. The expression levels of type I collagen in muscle remained constant across all groups (Supplemental Fig. 2f), suggesting that the observed recovery occurred within the femur tissue.

Furthermore, we investigated whether JTT might exacerbate ectopic calcification, a common feature of klotho-deficient models (Voelkl et al., 2013). We examined the expression of genes associated with vascular calcification in the femoris muscle. While the expression of the phosphate transporter *Pit1* (also known as *Slc20a1*) was significantly increased in KL+ mice, other key osteogenic master regulators and functional markers, such as *Runx2*, *Sp7*, *Alpl*, *Bglap*, *Bmp2*, *Msx2* and *Spp1*, showed no such increase (Supplemental Fig. 4). These findings suggest that the JTT-induced activation of the osteogenic program is specific to the bone microenvironment.

Collectively, these results suggest that JTT promotes bone formation primarily by increasing the expression of genes involved in collagen synthesis.

### 3.6 Expression of osteoclast differentiation - related genes is restored by JTT

The micro-CT analysis revealed that JTT treatment influenced cortical bone structure; specifically, it increased both cortical bone mass (e.g., Ct.V and Ct. V/Tr.Ar) and cortical porosity (Po.V and Ct.Po), as shown in Fig. 1. Indeed, genes showing a V-shaped recovery after JTT administration included those critical for osteoclast differentiation and activity (Fig. 4b left). Osteoclast differentiation requires the proliferation of monocyte/macrophage lineage osteoclast precursor cells induced by macrophage colony-stimulating factor (M-CSF), the RANK-RANKL pathway, and downstream signaling pathways such as the NF-κB pathway and MAPK pathway (Negishi-Koga and Takayanagi, 2009; Liu and Zhang, 2015). Furthermore, osteoclast-associated receptor (OSCAR) of the immunoglobulin-like receptor and the Fc receptor γ pathway (FcRγ) are crucial for osteoclast formation (Shinohara et al., 2008; Takegahara et al., 2024). These pathways activate differentiation by inducing nuclear factor of activated T cells, c1 (NFATc1), a master transcription factor for osteoclastogenesis (Shinohara et al., 2008; Takegahara et al., 2024).

**Fig. 5:**
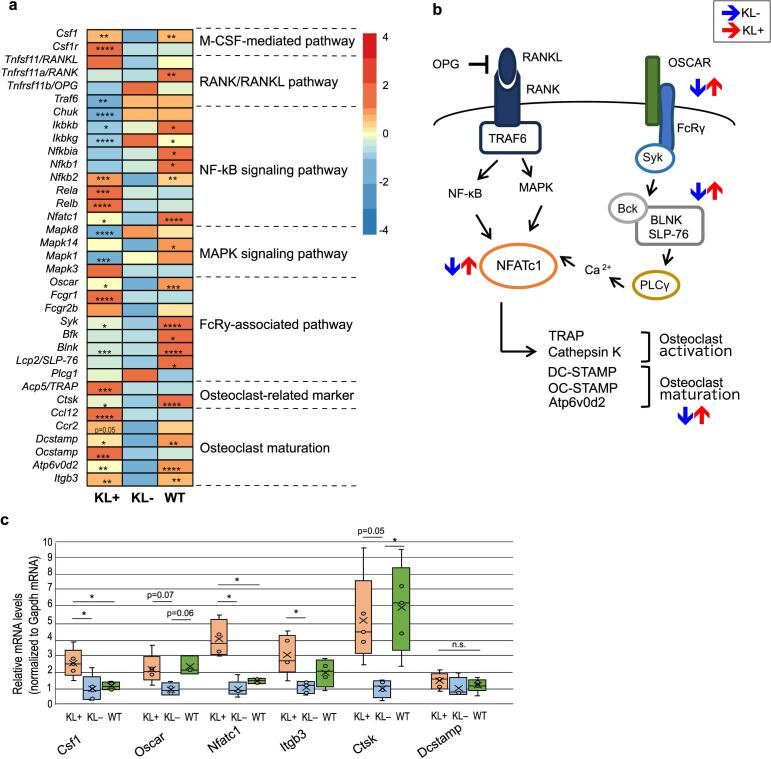
Altered osteoclast differentiation pathways in femoral samples and their recovery by JTT. (a) Heatmap of the expression levels of genes associated with osteoclast differentiation and maturation-related pathways. *P < 0.05, **P < 0.01, ***P < 0.001, ****P < 0.0001 versus KL− mice, according to the likelihood ratio test. (b) Schematic diagram of osteoclast differentiation and maturation. RNAKL and RNAK binding promote downstream NF-κB and MAPK signaling via adapter proteins, including TRF6, leading to increased NFATc1 levels. Osteoprotegerin (OPG) inhibits the RANKL-RANK interaction and negatively regulates bone resorption. Fc receptor gamma chain (FcRγ), a costimulatory molecule for RANK signaling, binds to the cell surface receptor osteoclast-associated receptor (OSCAR) and increases NFATc1 expression via phospholipase Cγ (PLCγ)-mediated Ca^2+^ signaling. NFATc1 induces genes essential for osteoclast activity, such as TRAP and cathepsin K. NFATc1 induces the expression of molecules involved in osteoclast maturation. In KL− mice, the expression levels of FcRγ signaling-related genes, *Nfatc1*, and osteoclast maturation-related genes decreased (blue arrows), whereas JTT administration restored the expression of these genes (red arrows). (c) mRNA expression levels of *Csf1*, *Oscar*, *Nfatc1*, *Itgb3*, *Ctsk* and *Dcstamp* measured by RT-qPCR are shown as the fold change relative to those in KL− mice (means ± SDs, n = 4–5). Box plots display the 25th and 75th percentiles (bottom and top of the box, respectively), the horizontal line within the box represents the median, crosses indicate the mean, and whiskers represent the minimum and maximum values. *Gapdh* was used as an internal control. n.s., not significant; *P < 0.05 (statistical tests were performed as described in the Methods).

Most genes encoding RANKL (*Tnfsf11*), RANK (*Tnfrsf11a*), members of the tumor necrosis factor receptor-associated factor (TRAF) family that mediate RANK signaling, and osteoprotegerin (OPG/*Tnfrsf11b*), which contributes to the suppression of osteoclast differentiation, showed few significant changes in KL− mice or after JTT treatment (Fig. 5a). However, when the RNAKL/OPG ratio, which is widely used for evaluating bone resorption (Domazetovic et al., 2017; Abid et al., 2023), was calculated on the base of gene expression levels, the ratio in KL+ mice was greater than that in both KL− mice and WT mice, suggesting that JTT may promote osteoclast activation. This occurred because compared with KL− mice, KL+ mice had increased Tnfsf11 expression (mean read count: 431.6 ± 161.7 vs. 363.7 ± 316.2), whereas Tnfrsf11b expression decreased in KL+ mice (354.6 ± 371.8 vs. 714.2 ± 564.9) (Table S3). The average RANKL/OPG ratios for individual KL+, KL−, and WT mice were approximately 2.28, 0.55, and 0.99, respectively (data not shown).

The expression of the NF-κB transcription factor family (*NF-κB2* and *RelB*), downstream targets of RANK (Sun, 2011), and its target factor NFATC1 was restored by JTT administration (Fig. 5a). Genes in the FcRγ-associated pathway (*Oscar*, *Syk*, and *Blnk*) also trended to increase in expression in response to JTT administration. Moreover, among the genes that showed a V-shaped recovery, several genes related to osteoclast activation and maturation regulated by NFATc1, including genes encoding cathepsin K (*Ctsk*), β3 integrin (*Itgb3*), and DC-STAMP (*Dcstamp*), were detected (Figs. 4b, 5a) (Negishi-Koga and Takayanagi, 2009). Additionally, genes essential for the formation of multinucleated osteoclasts, including Ccl2 (*Ccl12*) and its receptors (*Ccr2*), *Ocstamp*, and *Atp6v0d2* (Takegahara et al., 2024), also increased in expression following JTT treatment (Fig. 5a), suggesting that these pathways contribute to the JTT-mediated activation of osteoclast differentiation (Fig. 5b). The RNA expression levels of several of these genes were confirmed by qRT-PCR (Fig. 5c). Although changes in *Dcstamp* expression were not detected, the expression of *Csf1*, *Oscar*, *Nfatc1*, *Ctsk*, and *Itgb3* increased in response to JTT compared with that in KL− mice, which is largely consistent with the recovery trends suggested by the results of the RNA-seq analysis.

Taken together, these findings indicate that JTT upregulates key genetic pathways involved in both bone formation and osteoclast differentiation, which may underly the observed changes in cortical bone volume and cortical porosity in klotho mice.

### 3.7 JTT regulates the derepression of Wnt signaling

Many bone-related genes were detected among the reverse V-shaped recovery genes shown in Fig. 4b. Notably, this group contained numerous genes associated with the Wnt signaling pathway and the bone morphogenetic protein (BMP) signaling pathway, both of which are crucial for bone formation and homeostasis and promote multiple stages of osteoblast differentiation (Song et al., 2021; Hu et al., 2024).

**Fig. 6:**
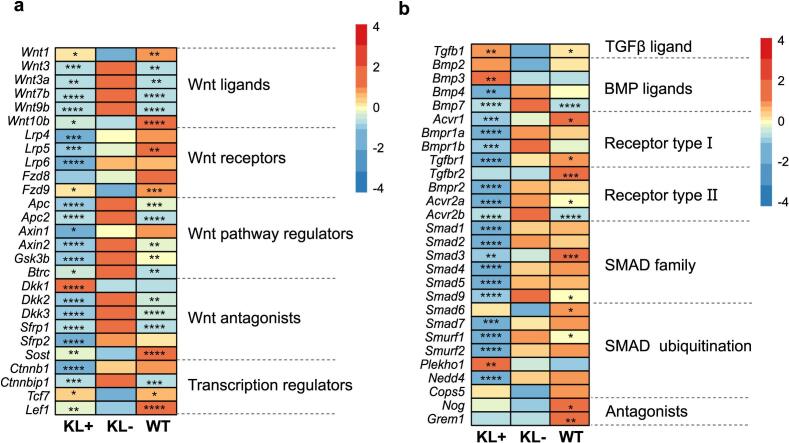
Altered Wnt signaling in femoral samples and their recovery by JTT. (a) Heatmap of the expression levels of genes associated with Wnt/β-catenin signaling. (b) Heatmap of the expression levels of genes associated with the bone morphometric protein (BMP)-suppressor of mothers against the decapentaplegic homolog (SMAD) signaling pathway. In (a), (b), *P < 0.05, **P < 0.01, ***P < 0.001, ****P < 0.0001 vs. KL− mice, according to the likelihood ratio test.

We therefore examined representative genes involved in canonical Wnt signaling (Fig. 6a). Several negative regulators of the Wnt pathway (*Apc1*, *Apc2*, *Axin2*, *Gsk3b*, *Btrc*, and *Ctnnbip1*) and Wnt antagonists (*Dkk2*, *Dkk3*, and *Sfrp1*) exhibited reverse V-shaped recovery, whereas the transcription factors *Tcf7* and *Lef1*, which act downstream of Wnt/β-catenin signaling, showed V-shaped recovery. In contrast, other Wnt antagonists, *Dkk1* and *Sost*, were upregulated by JTT, and several Wnt ligands and receptors were significantly downregulated following JTT administration. TGF-β and BMP signaling also regulate osteoblast differentiation, bone formation, and bone homeostasis via Smad-dependent pathways (Wu et al., 2016). When we examined the expression of gene related to TGF-β/BMP signaling in bone (Fig. 6b), the expression of genes encoding BMP ligands, receptors, and Smad family members did not consistently change in KL− mice and was not rescued by JTT; instead, the expression of most genes significantly decreased. *Tgfb1* expression showed a V-shaped recovery after treatment with JTT.

These findings suggest that JTT treatment induces a transcriptional shift potentially promoting the derepression of Wnt signaling and the re-establishment of negative feedback loops, which may contribute to the restoration of osteoblast differentiation and bone formation.

### 3.8 Neural signaling-related gene expression is restored by JTT

As described above, JTT increased the expression of bone-related genes. Interestingly, many of the biological processes enriched in the reverse V-shaped recovery genes were associated with neuronal regulation, such as synapse transmission and synapse organization (Fig. 3f). Consistently, KEGG pathway analysis revealed significant enrichment of these genes in multiple neurotransmission systems, including glutamatergic, GABAergic, dopaminergic, cholinergic, adrenergic, and circadian rhythm pathways (Table S2). It has been reported that both osteoblasts and osteoclasts express adrenergic and neuropeptide receptors, and that Increased sympathetic activity suppresses bone formation (Takeda et al., 2002; Togari et al., 2012).

A heatmap of several neurotransmission-related genes is shown in Supplementary Fig. 5, which demonstrates that their expression was markedly elevated in KL− bone and suppressed in KL+ bone. Interestingly, both excitatory and inhibitory neurotransmission pathways were uniformly elevated in KL− mice. This pattern suggests that neurotransmission pathways may be dysregulated in KL− mice. Circadian rhythm genes, which influence bone remodeling and are induced by β-adrenergic receptor signaling in osteoblasts (Komoto et al., 2012; Hirai et al., 2014), were also upregulated in KL− mice and downregulated by JTT administration.

In the brain, downregulation of Klotho has been linked to increased neurodegeneration driven by glutamate toxicity and β-amyloid accumulation, and evidence that Klotho itself exerts neuroprotective effects is growing (Kuang et al., 2014; Abraham et al., 2016; Torbus-Paluszczak et al., 2018). We therefore examined the expression of the same neurotransmission-related genes in whole-brain tissue, but the expression patterns varied, and no clear effects of JTT were observed (Supplementary Fig. 5). The peripheral and central nervous systems may respond to JTT differently, and more detailed analysis will require regional dissection of the brain.

### 3.9 JTT increases the expression of genes encoding antioxidant enzymes in the femur

Many bone diseases, including osteoporosis, are linked to oxidative stress. Reactive oxygen species induce osteoclastogenesis and inhibit mineralization and osteogenesis. Antioxidants resist these reactive oxygen species and contribute to bone formation (Domazetovic et al., 2017; Ren et al., 2021). The antiaging gene *Klotho* has also been reported to increase both antioxidant activity and resistance to oxidative stress (Yamamoto et al., 2005; Lim et al., 2017). In addition, many Kampo formulations possess antioxidant properties, and several herbal ingredients in JTT have been reported to exhibit antioxidant activity (Astragalus root, peony root, and ginseng (Zhao et al., 2020); Rehmannia root (Huang et al., 2024); and Glycyrrhiza (Sohail et al., 2018)). We therefore examined the effects of JTT on antioxidant enzymes in bone tissue.

**Fig. 7:**
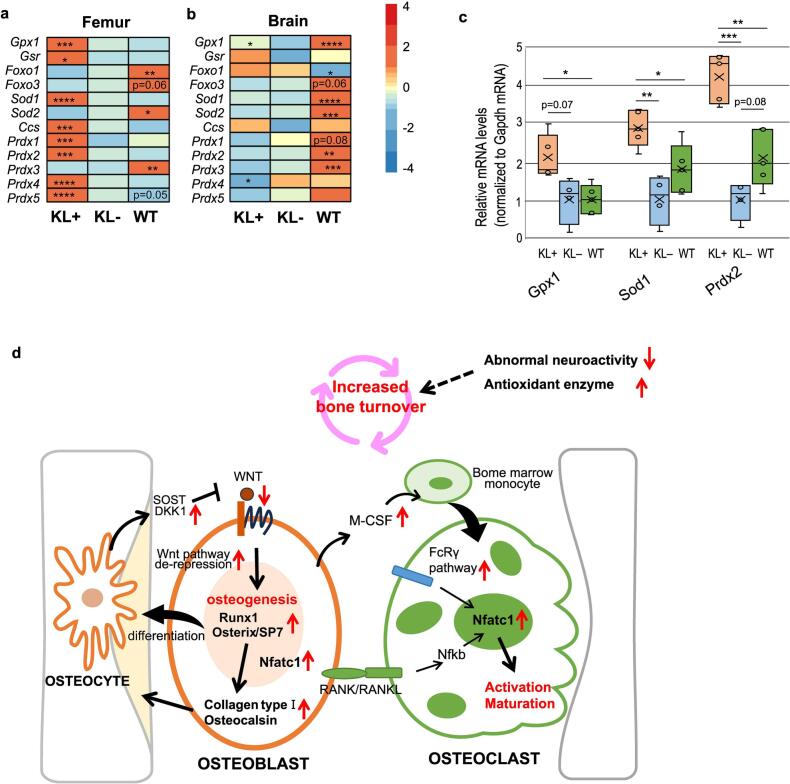
JTT upregulates the expression of genes encoding antioxidant enzymes in femoral samples. (a) Heatmap of the expression levels of genes encoding antioxidant enzymes in the femur. (b) Heatmap of the expression levels of genes encoding antioxidant enzymes in the brain. In (a), (b), *P < 0.05, **P < 0.01, ***P < 0.001, ****P < 0.0001 vs. KL− mice, according to the likelihood ratio test. (c) mRNA expression levels of *Gpx1*, *Sod1*, and *Prdx2* measured by RT-qPCR are shown as the fold change relative to those in KL− mice (means ± SDs, n = 4–5). Box plots display the 25th and 75th percentiles (bottom and top of the box, respectively), crosses indicate the mean, and whiskers represent the minimum and maximum values. *Gapdh* was used as an internal control. *P < 0.05, **P < 0.01, ***P < 0.001 (statistical tests were performed as described in the Methods). (d) Schematic representation of the integrated hypothesis for how JTT restores bone remodeling. Red arrows indicate the predicted direction of JTT action. Osteoblast-derived macrophage colony-stimulating factor (M-CSF) promotes osteoclast differentiation. JTT increases the expression of M-CSF and FcRγ-related signaling genes, contributing to osteoclast activity and maturation via increased NFATc1 expression. In osteoblasts, JTT promotes Osterix (*Sp7*) and *Runx1* expression through the derepression of Wnt signaling, and Osterix together with NFATc1 induces type I collagen expression and ossification. Osteocytes secrete the Wnt inhibitors SOST and DKK1, which negatively regulate osteoblastic differentiation. JTT also modulated the expression of nervous system-associated genes and promoted the expression of genes encoding antioxidant enzymes in KL− femurs, suggesting a potential mitigation of excessive bone resorption was suppressed.

We analyzed the expression of genes encoding major antioxidant enzymes in the femurs of klotho mice. The expression of glutathione peroxidase (*Gpx1*), glutathione reductase (*Gsr*), superoxide dismutase 1 (*Sod1*), the copper chaperone for SOD (*Ccs*), and peroxiredoxins (*Prdx1*, *Prdx2*, *Prdx4*, and *Prdx5*) did not significantly change in KL− mice compared with WT mice, but their expression levels significantly increased following JTT administration (Fig. 7a). qRT-PCR analysis verified the RNA-seq results for *Gpx1*, *Sod1*, and *Prdx2* expression (Fig. 7c). In contrast, the expression of the forkhead transcription factors *Foxo1* and *Foxo3*, as well as *Sod2*, was significantly lower in KL− mice than in WT mice, and JTT did not restore their expression (Fig. 7a). Because FOXOs upregulate manganese superoxide dismutase (MnSOD/*Sod2*) and Klotho has been reported to activate FOXOs (Yamamoto et al., 2005; Lim et al., 2017), these findings suggest that JTT induces the expression of genes encoding antioxidant enzymes through a pathway independent of the Klotho–FOXO–Sod2 axis.

In whole-brain tissue, most genes encoding antioxidant enzymes were expressed at lower levels in KL− mice than in WT mice, and JTT did not significantly increase their expression (Fig. 7b). Collectively, these results indicate that JTT selectively induces antioxidant enzyme-encoding gene expression in bone tissue.

## 4 Discussion

Osteoporosis increases fracture risk and is a major contributor to care dependency among older adults. Klotho-deficient mice exhibit low bone remodeling activity and reduced numbers of osteoclasts and osteoblasts, resulting in an osteoporosis-like phenotype (Kuro-o et al., 1997; Kawaguchi, 2006). Although this phenotype similar to that of age-related osteoporosis in terms of low turnover in males, Klotho deficiency is characterized by excessive renal vitamin D release leading to hypercalcemia and hyperphosphatemia (Erben and Andrukhova, 2017). This differs from senile osteoporosis, which is driven by vitamin D and calcium deficiency, reduced VDR expression, and elevated PTH levels (Compston et al., 2019; Horst et al., 1990; Bhattarai et al., 2020). Therefore, while the Klotho-deficient model provides invaluable insights into the molecular pathways of low-turnover bone aging, caution must be exercised when extrapolating these systemic mechanism-based findings directly to the clinical pathophysiology of typical human senile osteoporosis.

Excessive amounts of bioactive vitamin D can promote osteoclast-mediated bone resorption via osteoblastic VDR and increase serum Ca and FGF23 levels (Mori et al., 2020). Thus, the abnormal increase in *Vdr* expression induced by JTT in this study is unlikely to contribute to mineral metabolism or bone formation, and the improvement in BMD in KL+ mice was likely not mediated by vitamin D-related pathways. Although serum FGF23 levels typically correlate with *Fgf23* mRNA expression in bone and other tissues (Hassan et al., 2016; Leifheit-Nestler et al., 2018), KL+ mice in this study exhibited significant BMD improvement despite maintaining elevated *Fgf23* and *Vdr* mRNA levels. Furthermore, given that bioactive FGF23 fails to reduce serum phosphate in the absence of Klotho (Nakatani et al., 2009; Razzaque, 2009), our findings suggest that, under conditions of Klotho deficiency, JTT exerts its beneficial effects on the skeleton independently of the FGF23–Klotho–vitamin D axis. This interpretation is supported by findings in SAMP6 senile osteoporosis mice, where JTT prevented bone loss and reduced serum Ca, P, and PTH levels (Chen et al., 2005), suggesting that the beneficial effects of JTT on mineral metabolism may depend, at least in part, on an intact Klotho–FGF23 axis.

Although Klotho is expressed mainly in the kidney and brain and its deficiency causes systemic premature aging (Kawaguchi, 2006), JTT did not restore gene expression in either tissue in this study. These findings indicate that JTT may act independently of Klotho-mediated pathways and directly affect bone formation.

JTT is a nourishing agent that was originally used for improving disruptions and imbalances in the homeostatic condition of the body (Saiki, 2000). In this study, we demonstrated that JTT effectively increases the BMD and clarified the mechanisms by which it ameliorates cortical bone metabolism in Klotho-deficient mice, which exhibit osteoporosis-like phenotypes characterized by low bone remodeling, as follows: (i) restoration of the expression of genes required for collagen synthesis and osteoblast differentiation, (ii) restoration of the expression of genes involved in osteoclast differentiation and maturation, (iii) suppression of the aberrant overexpression of peripheral neurotransmission-related genes, and (iv) increased expression of antioxidant enzymes.

### 4.1 Nfatc1 is a central mediator of JTT-induced bone formation and osteogenic differentiation

RNA-seq indicated that JTT may have multiple effects on bone formation, osteoblastic differentiation, and osteoclastic differentiation. In terms of bone formation, many collagen-related genes exhibited a V-shaped recovery in expression. JTT also increased the expression of Tgfb1 and the Wnt signaling transcription factors TCF/LEF, which are involved in the promotion of collagen synthesis, while it reduced the expression of several Wnt regulators and antagonists (Fig. 4, Fig. 5). The genes related to collagen synthesis that exhibited restored expression in this study included those that are known markers of osteogenesis imperfecta (OI). OI is characterized by reduced bone quality and bone mass, leading to an increased risk of fractures (Boudin and Van Hul, 2017; Busse et al., 2024), supporting the central role of increased collagen synthesis in bone matrix formation. Although OI is caused by genetic mutations and JTT is unlikely to have direct therapeutic relevance for this disorder, the present findings raise the intriguing possibility that JTT may have applications beyond osteoporosis, potentially benefiting other conditions associated with bone fragility.

The Wnt signaling pathway promotes the transcription of osterix (SP7) via β-catenin, and osterix induces bone formation by modulating the expression of type I collagen and osteocalcin (Liu et al., 2020). Noncanonical Wnt/Ca^2+^ signaling has also been reported to activate nuclear factor of activated T cells (NFAT) (Hu et al., 2024; Bengoa-Vergniory and Kypta, 2015). NFATC1 is a master regulator of osteoclast activation and is activated through RANKL–RANK signaling and the FcRγ pathway (Takegahara et al., 2024). In addition, osterix and NFATC1 can form a complex that induces type I collagen expression, indicating that NFATC1 also contributes to bone formation (Liu et al., 2020). In this study, JTT restored the reduced expression of *Nfatc1* in klotho mice. Furthermore, JTT promoted the expression of upstream factors associated with FcRγ and those in the NF-κB signaling pathway, as well as downstream genes related to type I collagen, osteoclast activation and maturation (Fig. 4, Fig. 5). Together, these findings suggest that JTT may promote not only osteoclast differentiation but also bone formation through the restoration of *Nfatc1* expression.

### 4.2 Restoration of the bone formation negative feedback loop by JTT

While JTT promotes bone formation and osteoclast differentiation, the RNA-seq data also indicated that it suppresses osteoblast activity. For example, several Wnt ligands and receptors were significantly downregulated by JTT treatment (Fig. 6a). Osterix promotes osteoblastic differentiation, but it also negatively regulates Wnt signaling in osteoblasts through the induction of the secretion of SOST and DKK1 from osteocytes (Liu et al., 2020). In this study, the recovery of Osterix expression by JTT was accompanied by increased expression of *Sost* and *Dkk1*, suggesting that the negative feedback loop of Wnt signaling may have resumed function because the osteocytes recovered. In contrast, the levels of other Wnt antagonists, such as *Dkk2*, *Dkk3*, *Sfrp1*, and *Sfrp2*, were significantly decreased by JTT, resulting in a mixed pattern of Wnt activation and inhibition. Unlike DKK1 and SOST, which are mainly secreted by osteocytes, DKK2 is expressed at higher levels in osteoblasts than in osteocytes and contributes to osteogenic differentiation (Li et al., 2005). sFRPs are also predominantly expressed in osteoblasts and bone marrow (Nakajima et al., 2009). Furthermore, JTT downregulated many BMP ligands, receptors, and Smad family genes essential for osteoblast differentiation (Fig. 6b), which initially appears to contradict the observed improvement in bone density. However, this decrease in BMP signaling may reflect a compensatory response in which excessive osteoblast formation is suppressed as osteoblasts differentiate into osteocytes. Importantly, it should be noted that Wnt signaling activity is extensively regulated by post-translational modifications, including phosphorylation and protein complex formation. While our RNA-seq analysis indicates a concerted transcriptional shift towards a permissive state for Wnt signaling and a potential restoration of feedback inhibition, further investigations, such as protein-level assays, will be required to confirm the functional activation of this pathway in vivo. Finally, as our RNA-seq analysis was performed on whole bone tissue, changes in cellular composition induced by JTT may have influenced the gene expression profiles. For example, an increased proportion of osteocytes could augment the increase in *Sost* and *Dkk1* expression, whereas the relative decreases in the expression levels of osteoblast-derived Wnt antagonists (*Dkk2* and *Sfrp1,2*) might be more pronounced. Future studies employing single-cell analyses or cell type-specific expression profiling will help clarify the precise cellular targets and mechanisms of JTT.

### 4.3 Modulation of the peripheral nervous system by JTT in bone protection

RNA-seq analysis of the femur revealed that JTT significantly modulated a subset of nervous system-associated genes that were aberrantly altered in KL− mice. Although their exact functional relevance has not yet been fully elucidated, accumulating evidence indicates that a direct network between peripheral nerve fibers and bone cells is essential for regulating bone metabolism in vivo (Togari et al., 2012).

Adrenergic and neuropeptide receptors are present on bone nerve fibers, and both osteoblasts and osteoclasts express these receptors. Importantly, increased sympathetic activity reduces bone mass by increasing bone resorption and decreasing bone formation (Takeda et al., 2002; Togari et al., 2012). For example, stimulation of β-adrenergic receptors suppresses bone formation by reducing osteoblast number and type I collagen expression in osteoblasts (Takeda et al., 2002).

These findings suggest that excessive sympathetic nervous system activation contributes to severe bone loss in KL− mice. By counteracting these abnormal transcriptional changes, JTT may alleviate this overactivation and thereby rescue bone formation. Although further research is needed to validate this neural-skeletal axis, our findings indicate that the therapeutic effects of JTT likely involve the regulation of neuro-osseous crosstalk.

### 4.4 Induction of antioxidant enzyme-encoding genes by JTT

Oxidative stress increases with aging, menopause, and lifestyle-related diseases, promoting bone resorption and reducing bone strength. In contrast, antioxidants have been reported to promote osteoblast differentiation and suppress osteoclast activity (Domazetovic et al., 2017; Ren et al., 2021). Klotho also increases oxidative stress resistance by inducing *Sod2* gene expression through the activation of FoxO transcription factors (Yamamoto et al., 2005; Lim et al., 2017). Zeldich et al. (Zeldich et al., 2014) demonstrated that Klotho induces the thioredoxin/peroxiredoxin redox system, particularly Prx2, in hippocampal neurons, suggesting that Klotho deficiency may increase host susceptibility to oxidative stress.

Interestingly, JTT significantly increased the expression of several genes encoding antioxidant enzymes, including Gpx1, Sod1, and Prdx2, in bone tissue but had no effect on the Klotho–FoxO–Sod2 axis (Fig. 7). These findings suggest that JTT induces antioxidant responses through molecular pathways distinct from those mediated by Klotho. Several herbal components in JTT have been shown to have antioxidant properties (Zhao et al., 2020; Huang et al., 2024; Sohail et al., 2018), and its multicomponent nature may contribute to the selective induction of antioxidant enzymes in bone tissue.

In contrast, no improvement was observed in the whole brain. This may be due to region-specific gene expression changes being diluted in whole-brain analyze, or because the effects of JTT are weaker in the central nervous system than in bone or involve different regulatory pathways. Therefore, region-specific brain analysis or cell type-specific analyses will be necessary to clarify the tissue-specific actions of JTT.

JTT selectively induces the expression of genes encoding antioxidant enzymes in bone tissue, potentially compensating for the increased susceptibility to oxidative stress associated with Klotho deficiency. This action is considered one of the important mechanisms contributing to improved bone remodeling and maintenance of bone quality.

### 4.5 Immune- and muscle-related gene expression was restored by JTT

JTT also restored the expression of immune-related and muscle-related genes (Fig. 3e). The fatty acids contained in JTT (oleic acid and linoleic acid) have been reported to increase immune activity by promoting the proliferation of mouse hematopoietic stem cells (Hisha et al., 1997) and bone marrow cells from patients with Shwachman–Diamond syndrome, a condition characterized by bone marrow failure and skeletal abnormalities (Hisha et al., 2002). Because the immune system is closely linked to bone remodeling (Clowes et al., 2005), it is intriguing to speculate that the recovery of immune-related gene expression observed after JTT administration may contribute to improved bone formation in klotho mice.

JTT is clinically used to treat frailty, and it has been reported to suppress skeletal muscle atrophy in aging and diabetic mouse models (Morita et al., 2021; Ishida et al., 2022). Although how the recovery of muscle-related gene expression observed in bone marrow and bone tissue in this study influences bone formation remains unclear, it is well recognized that sarcopenia-associated muscle loss and frailty in older adults are closely linked to osteoporosis and fracture risk (Committee for Developing Guidelines for the Prevention and Treatment of Osteoporosis, 2025; Oliveira and Vaz, 2015). These findings underscore the importance of early prevention and intervention.

## 5 Limitations of the present study

The findings of this study should be interpreted in light of several limitations. First, the greatest limitation is that many of the conclusions were inferred primarily from RNA-seq data. Because key signaling pathways are extensively regulated by post-translational modifications including phosphorylation and protein complex formation, our transcriptional data provide an indirect assessment of pathway modulation rather than direct evidence of functional protein activity. This reliance on transcriptional profiling also affects the interpretation of our microstructural findings; while the concurrent activation of bone formation and resorption pathways strongly suggests a JTT-mediated reactivation of bone remodeling, the lack of direct histological or functional evidence means that we cannot entirely exclude the possibility that the increased cortical pore volume partly reflects age-associated cortical porosity or localized deterioration. To fully demonstrate that JTT improves bone metabolism through multiple pathways, future studies will require complementary analyses, including detailed bone histomorphometry, gene and protein expression and hormone and mineral measurements, in not only bone, muscle, and the kidney but also in other relevant organs such as the intestine and blood.

Second, as noted above, the use of Klotho-deficient mice presents inherent limitations. Klotho deficiency affects bone metabolism, causing low bone remodeling osteoporosis (Kuro-o et al., 1997; Kawaguchi, 2006), whereas bone-specific Klotho deficiency has been reported to increase bone mass (Komaba et al., 2017). These findings highlight the complexity of the role of Klotho in skeletal homeostasis. Although the results of the present study revealed that JTT improves both bone formation and bone resorption, suggesting a capacity to restore disrupted homeostasis, validation in additional bone disease models is essential.

Third, the RNA-seq data obtained from femoral samples may contain mRNA derived from muscle and bone marrow. Although comparisons of expression patterns among the femur, bone marrow, and muscle suggest that muscle contamination is likely minimal, the possibility that tissue-specific gene changes were partially masked remains. Improvements in sample preparation methods and analytical techniques to minimize such contamination are necessary in future studies.

## 6 Conclusions

This study demonstrated that JTT ameliorates low bone remodeling in Klotho-deficient mice and increases bone mineral density through multiple coordinated mechanisms. JTT restored the expression of genes involved in collagen synthesis and osteoblast differentiation, normalized *Nfatc1*-centered pathways that regulate osteoclast maturation, suppressed aberrant peripheral nerve signaling, and increased the expression of antioxidant enzymes (Fig. 7d). These effects appear to occur independently of Klotho–FGF23 or vitamin D-related pathways, suggesting that JTT may influence skeletal homeostasis through alternative mechanisms. Together, our findings highlight the potential of JTT as a multitarget therapeutic approach for conditions associated with bone fragility and provide a foundation for future studies using additional disease models and tissue-specific analyses.

## CRediT authorship contribution statement

**Akiko Maruko:** Formal analysis, Investigation, Writing – original draft. **Kenshiro Oshima:** Data curation, Formal analysis, Writing – review & editing. **Akinori Nishi:** Data curation, Writing – review & editing. **Yoshinori Kobayashi:** Writing – review & editing. **Norihiro Okada:** Conceptualization, Supervision, Writing – review & editing.

## Ethics statement

All animal experiments, including breeding and dissections, were performed at CLEA Japan, Inc. (Tokyo), which is accredited by AAALAC International. All procedures were conducted in accordance with the Act on the Conservation and Sustainable Use of Biological Diversity through Regulations on the Use of Living Modified Organisms (Cartagena Act) and the Guide for the Care and Use of Laboratory Animals (ILAR guidelines), and were approved by the Institutional Animal Care and Use Committee of CLEA Japan.

## Declaration of Generative AI and AI-assisted technologies in the writing process

During the preparation of this work the authors used generative AI tools solely in order to improve the English expression and grammar of the manuscript. After using these tools, the authors reviewed and edited the content as needed and take full responsibility for the content of the published article.

## Declaration of competing interest

The authors AM, KO, YK, and NO received funding relevant to this research from TSUMURA & CO. The funder had the following involvement in the study: provision of Kampo formulations, information on Kampo composition and analysis, and interpretation of the data. Author AN was employed by TSUMURA & CO.

## Data Availability

RNA-seq datasets are deposited in the DDBJ Sequence Read Archive under accession numbers DRR908015-DRR908086, linked to the BioProject accession number PRJDB40256.
