# Supplementary material for: Bone transcriptomics reveals that juzentaihoto remodels and activates multiple pathways in Klotho-deficient mice
Source: Bone Rep. 2026 Jul 2;30:101936. doi: 10.1016/j.bonr.2026.101936 (PMC13355547; doi:10.1016/j.bonr.2026.101936)
Supplement: Supplementary file 1 — Supplementary figures [file mmc1.docx]

**Supplementary Information**


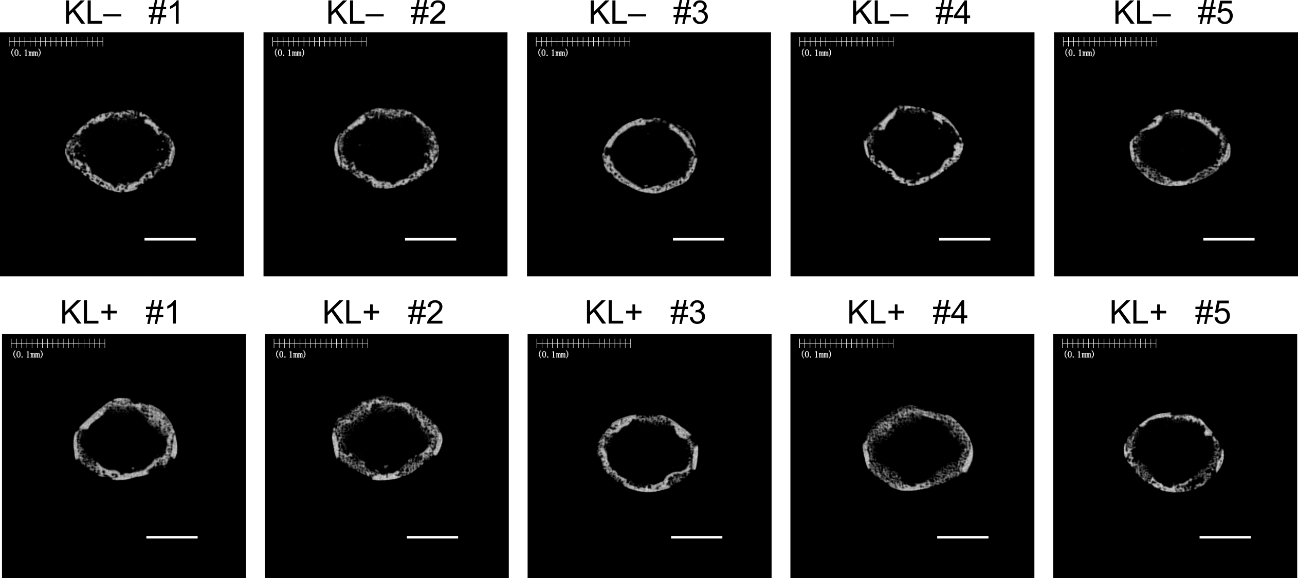


**Supplementary Figure 1. Micro-CT images of femurs in JTT-treated klotho mice (KL+) and untreated klotho mice (KL–)**

Micro-CT cross-sectional images of the distal diaphysis of the femur (central section B in Figure 1b). KL– #2 and KL+ #2 were used in Figure 1b. Bar = 1 mm.


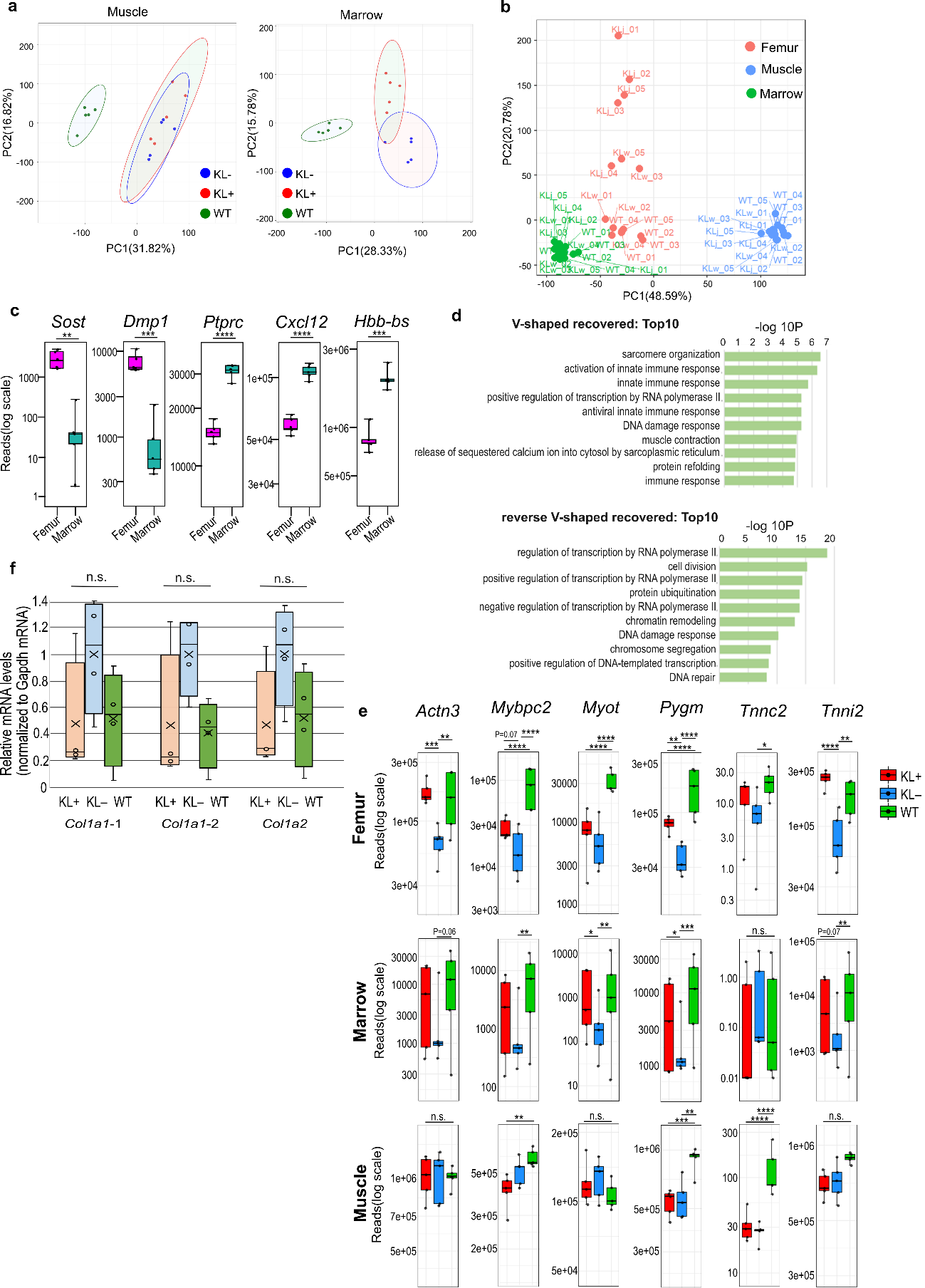


**Supplementary Figure 2.** **Transcriptomic comparison of femur, muscle, and bone marrow in KL–, KL+, and wild-type (WT) mice.**

(a) Principal component analysis (PCA) of muscle (left) and bone marrow (right) RNA-seq data from KL–, KL+, and WT mice. (b) PCA analysis of genes commonly expressed in the femur, muscle, and bone marrow. KL–, KL+, and WT mice (n=5) for each tissue were plotted, showing clear separation among the three tissues. (c) Box plots of tissue-specific marker gene expression levels in the femur and bone marrow samples of WT mice (n = 5). The y axis shows the read counts from RNA-seq data. Expression profiles are shown for osteocyte markers (*Sost* and *Dmp1*), a pan-hematopoietic/bone marrow marker (*Ptprc*), a bone marrow microenvironment marker (*Cxcl12*), and a hemoglobin gene (*Hbb-bs*). *P* values for the differential expression of genes were obtained using student’s t test; ***P* < 0.01, ****P* < 0.001, and *****P* < 0.0001. (d) The top 10 Gene Ontology (GO) biological process terms associated with V-shaped recovered genes (upper) and reverse V-shaped recovered genes (lower) in the bone marrow following JTT administration. (e) Box plot of expression levels of genes associated with the muscle‑specific (*Actn3, Mybpc2, Myot, Pygm, Tnnc2, Tnni2*) in the femur (Upper), bone marrow (middle), and muscle (lower). The *y* axis shows RNA-seq read counts. *P* values for the differential expression of genes were obtained using edgeR's likelihood ratio test; **P* < 0.05, ***P* < 0.01, ****P* < 0.001, and *****P* < 0.0001. (f) mRNA expression levels of *Col1a1_1, Col1a1_2*, and *Col1a2* measured by RT‑qPCR, shown as fold change relative to KL− (means ± SDs, n = 4–5) in muscle. Box plots display the 25th and 75th percentiles (bottom and top of the box, respectively), the horizontal line within the box represents the median, crosses indicate the mean, and whiskers represent the minimum and maximum values. *Gapdh* was used as an internal control. n.s., not significant (statistical tests were performed as described in the Methods).


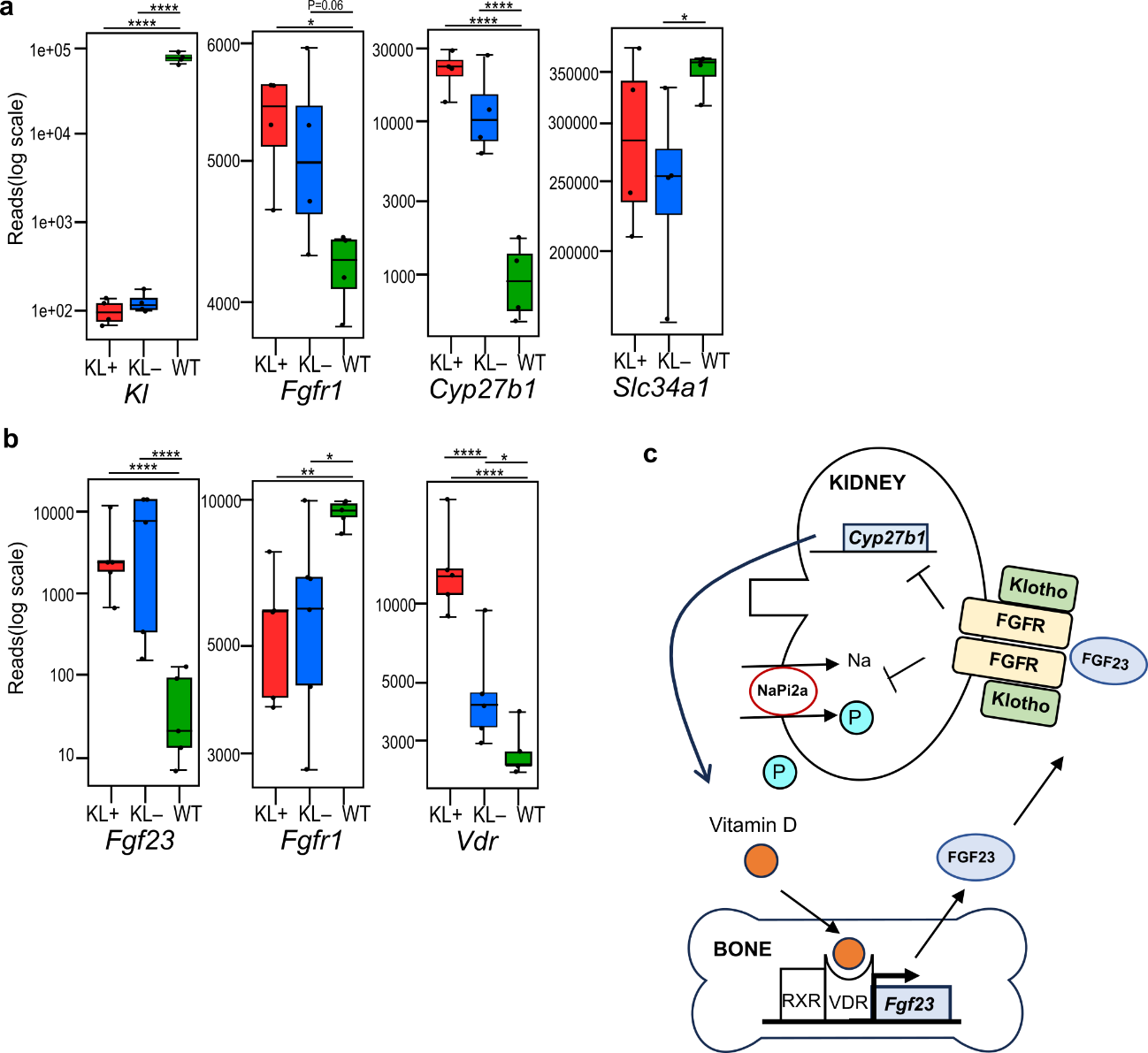


**Supplementary Figure 3. Expression of genes associated with the Klotho-FGF23 pathway in KL+, KL–, and WT mice.**

(a, b) Box plot of expression levels of genes associated with the Klotho and fibroblast growth factor 23 (FGF23) pathway in the kidney (n = 4) (a) and in the femur (n = 5) (b). The *y* axis shows the read counts from RNA-seq data. *P* values for the differential expression of genes were obtained using edgeR's likelihood ratio test; **P* < 0.05, ***P* < 0.01, and *****P* < 0.0001. (c) Klotho-FGF23 signaling in the kidney. Vitamin D binds to its receptor (VDR)/Retinoic acid X receptor (RXR) in bone, activating FGF23 expression. Circulating FGF23 binds to a receptor complex consisting of fibroblast growth factor receptor (FGFR) and Klotho in the kidney, leading to suppression of vitamin D synthesis and phosphate reabsorption.


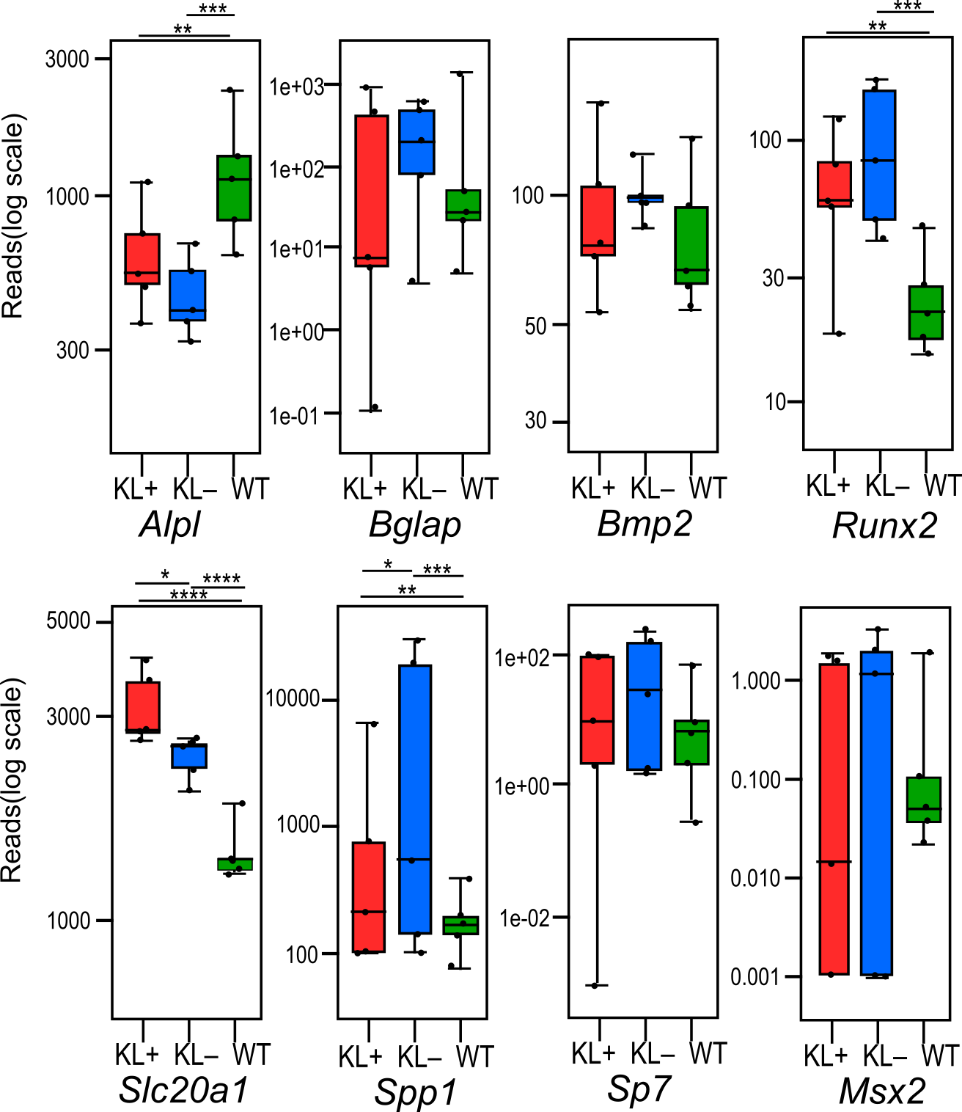


**Supplementary Figure 4. Expression of genes associated with vascular calcification in the femoral muscle of KL+, KL–, and WT mice.**

Box plot of expression levels of genes associated with the vascular calcification markers (*Alpl,* *Bglap*, *Bmp2*, *Runx2*, *Slc20a1*, *Spp1*, *Sp7*, and *Msx2*) in the femoral muscle (n = 5). The y axis shows the read counts from RNA-seq data. *P* values for the differential expression of genes were obtained using edgeR's likelihood ratio test; **P* < 0.05, ***P* < 0.01, and *****P* < 0.0001.


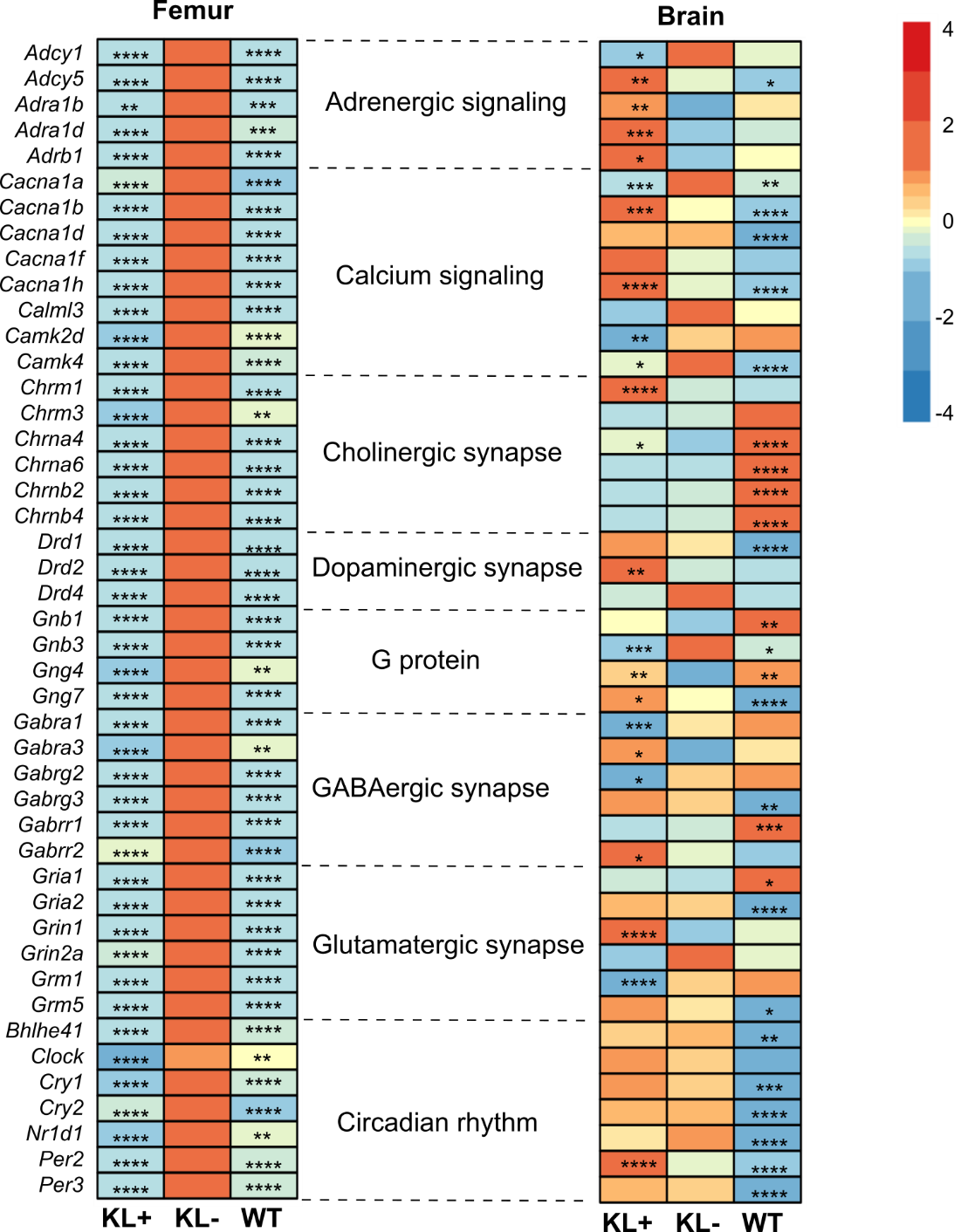


**Supplementary Figure 5. Femoral Neurotransmission-related genes and circadian clock-related genes that recovered in response to JTT**

Heatmap of the expression levels of genes associated with the neurotransmission-related genes in the femur (left) and in the brain (right). **P* < 0.05, ***P* < 0.01, ****P* < 0.001, *****P* < 0.0001 vs. KL− mice, according to the likelihood ratio test.
